# Iron–sulfur cluster redox chemistry and dimer dissociation in the outer mitochondrial membrane protein, mitoNEET

**DOI:** 10.1007/s00775-024-02093-7

**Published:** 2024-12-28

**Authors:** Kanita A. Chaudhry, Krishani K. Rajanayake, Richard T. Carroll, Dragan Isailovic, Max O. Funk

**Affiliations:** 1 Department of Chemistry and Biochemistry, University of Toledo, Toledo, OH USA; 2 Jacobs School of Medicine and Biomedical Sciences, University at Buffalo, Buffalo, NY USA; 3 Charles River Laboratories, Mattawan, MI USA; 4 Stow, OH USA

**Keywords:** Iron–sulfur cluster, Dithionite, mitoNEET, Electrospray ionization mass spectrometry (ESI–MS), Redox chemistry, Dimerization, Dissociation

## Abstract

The outer mitochondrial membrane protein known as mitoNEET was discovered when it was labeled by a photoaffinity derivative of the anti-diabetes medication, pioglitazone. The biological role for mitoNEET and its specific mechanism for achieving this remains an active subject for research. There is accumulating evidence suggesting that mitoNEET could be a component of mitochondrial FeS cofactor biogenesis. The protein was composed of an N-terminal membrane associated domain and a C-terminal domain oriented to the cytosol. The cytosolic domain was an iron–sulfur (2Fe–2S) metalloprotein with a rare 3Cys/1His coordination environment. It was previously reported that mitoNEET formed dimers that were remarkably sensitive to pH, likely a consequence of the protonation of the single His-iron ligand. The hypothesis pursued in the research reported here was that perhaps the dissociation of mitoNEET was also sensitive to the redox state of the iron sulfur cluster. To use native electrospray ionization mass spectrometry (ESI–MS) to monitor the reduction reaction ammonium dithionite was envisioned as the appropriate reagent to avoid sodium ion adduct formation from sodium dithionite. The preparation of ammonium dithionite was updated and the compound had the same properties as the sodium salt with redox dyes and the oxidized form of glutathione. The dissociation of mitoNEET treated with ammonium dithionite anaerobically was readily evident as ammonium dithionite was found to be compatible with redox chemistry evaluated by native ESI–MS.

**Graphical abstract:**

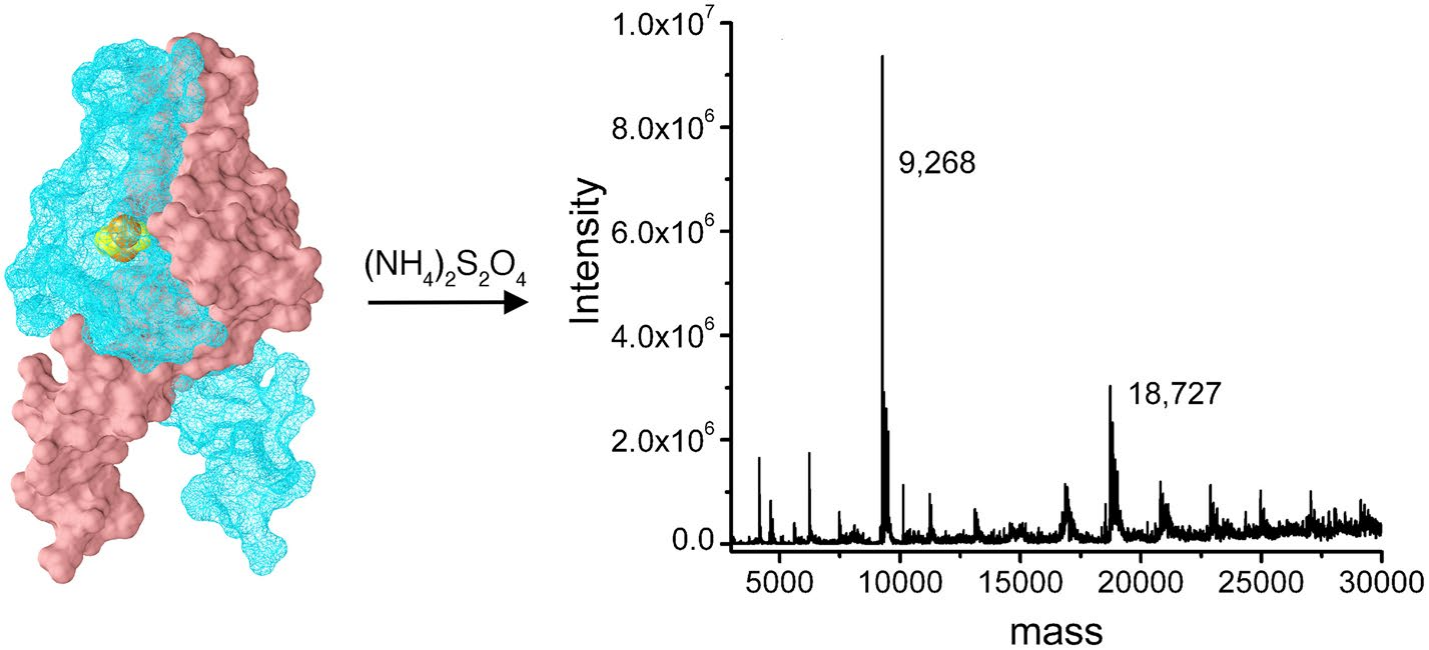

## Introduction

Investigations of redox biology involving metalloproteins require the means for altering the charge state of relevant metals in native proteins. Various spectroscopic measurements have been used to elucidate the consequences of these oxidation and reduction reactions. In some cases, direct electrochemical means can be applied for the conversions [1], while in others, chemical reagents have been employed. For example, sodium dithionite has been used extensively for biological reduction reactions [2]. While this is a decidedly non-physiological reductant, there are many examples in the literature for its application to the generation of specific charge states of metals in proteins and their subsequent characterization [3]. Whether dithionite accurately recapitulates the physiological consequences of reduction is a matter for ongoing research and discussion [4, 5].

The outer mitochondrial membrane-associated protein mitoNEET contains iron–sulfur clusters (2Fe–2S) ligated through a rare 3Cys/1His arrangement. mitoNEET was identified when it was labeled by a photoaffinity reagent based on the anti-diabetes drug pioglitazone [6]. Subsequently, the protein was linked to a number of pathological outcomes in cognitive impairment and cancer as well as in metabolic disorders [7]. The best evidence for a specific physiological function for mitoNEET has the protein acting as an intermediate in the biogenesis, e.g., direct transfer, of iron sulfur clusters taking place in mitochondria. For example, it was recently shown that iron sulfur clusters were passed from the inside of the organelle to outer mitochondrial membrane mitoNEET in a voltage-dependent anion channel-1 (VDAC-1) dependent process [8].

mitoNEET is anchored to the outer mitochondrial membrane by a single pass N-terminal sequence. The C-terminal cytosolic domain of human mitoNEET has been expressed in and isolated from *E. coli* and extensively characterized both spectroscopically and structurally via X-ray crystallography [9]. Each cytosol-directed C-terminal domain harbored a 2Fe–2S cluster and also contributed to an extensive dimerization interface. With regard to the 2Fe–2S clusters, for example, the oxidized form of the protein was EPR silent as a consequence of antiferromagnetic exchange coupling between the two iron(III) ions, whereas the electronic configurations and orientations of the sodium dithionite reduced 2Fe–2S clusters, iron(II)/iron(III), were thoroughly characterized based on their EPR properties [10, 11].

The dimeric form of the C-terminal domain of mitoNEET was found to be remarkably sensitive to solutions of low pH [12]. It was proposed that protonation of the single His ligand of the cluster, which is surface exposed in the three-dimensional structure, was responsible for this. According to native protein electrospray ionization mass spectrometry (ESI–MS), the dimer dissociated first at low pH followed by loss of the Fe–S cluster [13]. A subsequent study extended the characterization of the pH dependent stability of the Fe–S cluster in mitoNEET to include circular dichroism melting curves for site directed mutation derivatives incorporating amino acids in the vicinity of the cluster proposed to participate in hydrogen bonding networks [14]. The results indicated a close association between unfolding and loss of the cluster. Remarkably, the dithionite reduced form of the protein was found to be more stable at pH 5.2 than the oxidized form. When samples of mitoNEET with (holo) and without (apo) the FeS cofactor were analyzed by size exclusion chromatography, only dimers were observed for holo-mitoNEET and multimers for apo-mitoNEET. No monomers were detected in either case. The oligomeric status of the reduced protein was not reported.

The observed link between dissociation and pH prompted us to wonder if there might also be a relationship between dimerization and the redox state for the FeS cluster in the C-terminal domain. The application of sodium dithionite as a reductant to probe this possibility was considered likely to be problematic. The preparation of samples for ESI–MS requires removing sodium ions from the solutions to avoid the formation of adducts that form with proteins and complicate the measurements [15, 16]. In fact, we found the C-terminal domain of mitoNEET to be particularly susceptible to the formation of such adducts in the mass spectrometer (vide infra). The ammonium salt, i.e., ammonium dithionite was considered a logical alternative. Since the solutions already contained ammonium acetate as a volatile electrolyte, the presence of ammonium ions was not expected to interfere with the mass spectrometry.

## Results and discussion

**Fig. 1:**
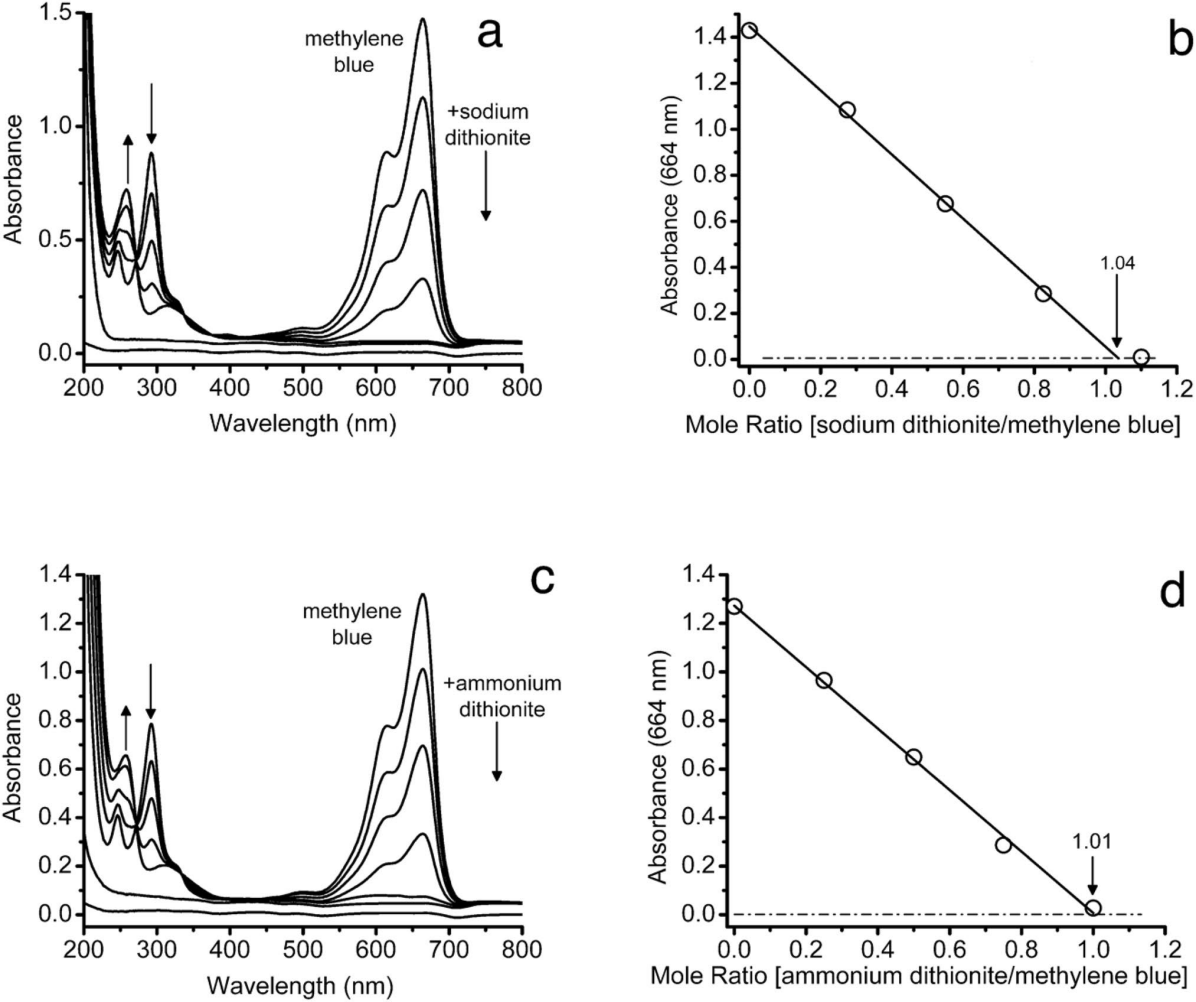
A comparison of the anaerobic titration of the redox dye methylene blue with sodium (**a**, **b**) and ammonium (**c**, **d**) dithionite in 10 mM ammonium acetate, pH 8.5 using UV–visible spectroscopy. **a** Methylene blue (21.17 µM, 2 mL) with sodium dithionite (2.912 mM, serial additions of 4 µL, i.e., 0, 4, 8, 12, 16 µL). **b** Moles sodium dithionite/moles methylene blue in 2 mL. Replication of equivalence points, 0.99 ± 0.04, *N* = 3. **c** Methylene blue (18.87 µM, 15 mL) with ammonium dithionite (35.35 mM, serial additions of 2 µL, i.e., 0, 2, 4, 6, 8 µL). **d** Moles ammonium dithionite/moles methylene blue in 15 mL. Replication of equivalence points, 0.96 ± 0.06, *N* = 5. The two bottom-most traces in both spectra were 10 mM ammonium acetate in a matched cuvette for absorbance correction and Ar, the atmosphere in the glove bag

Unlike the sodium salt, produced on an industrial scale, ammonium dithionite was not commercially available. In fact, there was only a single mention of its preparation in the Chemical Abstracts Service databases [17]. The preparation of the dithionite salts of potassium, rubidium, zinc and ammonium were described in the publication as reagents for the synthesis of metal fluorouranate (IV) compounds following by analogy the preparation of sodium dithionite starting with zinc reduction of sulfur dioxide and neutralization with the appropriate metal hydroxide. We found the results of isolations of solid ammonium dithionite as described to be inconsistent. Instead, the solution neutralized with ammonium hydroxide was immediately passed through a column of the ammonium form of Chelex 100 to remove any remaining traces of zinc (II), and was frozen on dry ice as aliquots after being purged with argon. The frozen solutions stored at −80 °C were suitable for reduction reactions once thawed and maintained in an inert atmosphere. The concentrations of ammonium dithionite solutions were determined spectrophotometrically using the molar extinction coefficient for the sodium salt at 315 nm, 8043 L/mol cm [18]. Ammonium dithionite quantitatively reduced redox dyes. As an example, a comparison for the reduction of methylene blue with sodium and ammonium dithionite salts is presented in Fig. 1. A linear and stoichiometric relationship was observed for the reaction between the dithionite salts and the dye.

**Fig. 2:**
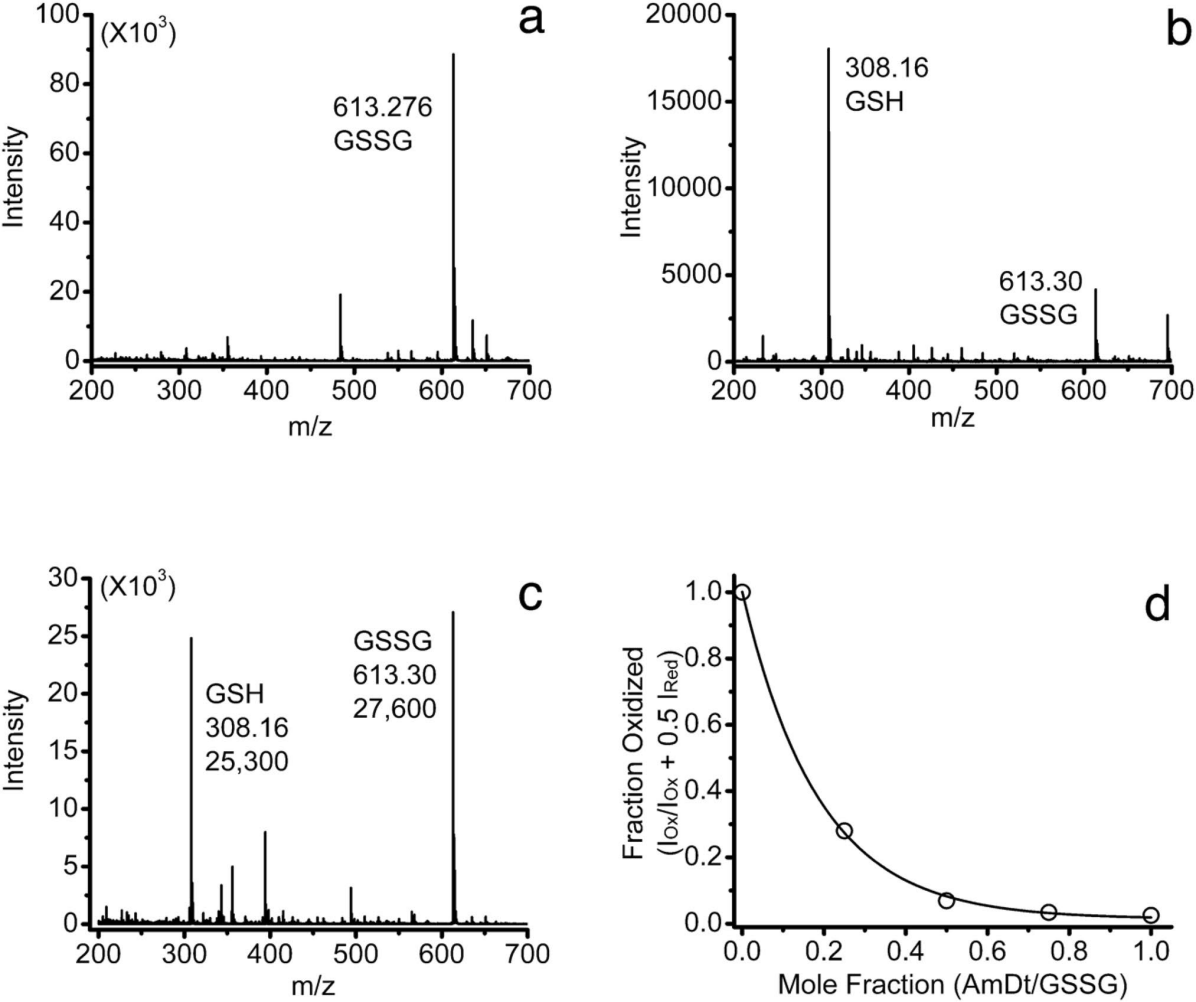
The anaerobic titration of oxidized L-glutathione with ammonium dithionite in 10 mM ammonium acetate, pH 8.5 using electrospray ionization mass spectrometry (ESI–MS). **a** Oxidized L-glutathione (43 µM, 10 mM ammonium acetate pH 8.5). **b** Oxidized L-glutathione (43 µM, 3 mL) combined with ammonium dithionite (5.34 mM, 0.012 mL, 0.5X GSSG) in 10 mM ammonium acetate pH 8.5. **c** Oxidized (43 µM) and reduced (43 µM) L-glutathione standards from commercial solids in 10 mM ammonium acetate pH 8.5. **d** Titration of oxidized L-glutathione (43 µM, 3.00 mL) with ammonium dithionite (5.34 mM, serial additions of 6 µL, i.e., 0, 6, 12, 18, 24 µL). The curve represents the fit of the data to a declining exponential function

The redox chemistry of glutathione was selected to test ammonium dithionite solutions for applications in ESI–MS. Treatment of the oxidized form of glutathione with 0.5 mol fraction of ammonium dithionite resulted in the consumption of significantly more than 50% of the oxidized glutathione (Fig. 2a, b). This was not a consequence of a difference in the sensitivity of the oxidized and reduced forms of glutathione in the mass spectrometer. When a sample consisting of a 50:50 mixture of the two substances prepared from commercial solids was analyzed, comparable intensities were observed (Fig. 2c). The treatment of oxidized glutathione with increasing amounts of ammonium dithionite consistently produced a non-linear reduction in the fraction oxidized versus the mole fraction of ammonium dithionite added while accounting for the 1:2 stoichiometry of the reaction and the roughly equivalent ionization efficiencies of the two species. The data presented in Fig. 2d were representative of four separate determinations. The curve fit was based on a declining exponential equation. These observations were consistent with an earlier finding initially overlooked for the reaction of oxidized glutathione with sodium dithionite [19]. The samples from the reaction mixture with the sodium salt were analyzed by RP-HPLC with UV detection at 215 nm. The publication [19] posited a catalytic process or a free-radical chain reaction to account for the behavior. In any case, the results of the current experiments and those presented in the literature provided compelling evidence that the two dithionite salts had the same redox properties with respect to glutathione and that the ammonium dithionite did not noticeably affect the ESI–MS measurements.

**Fig. 3:**
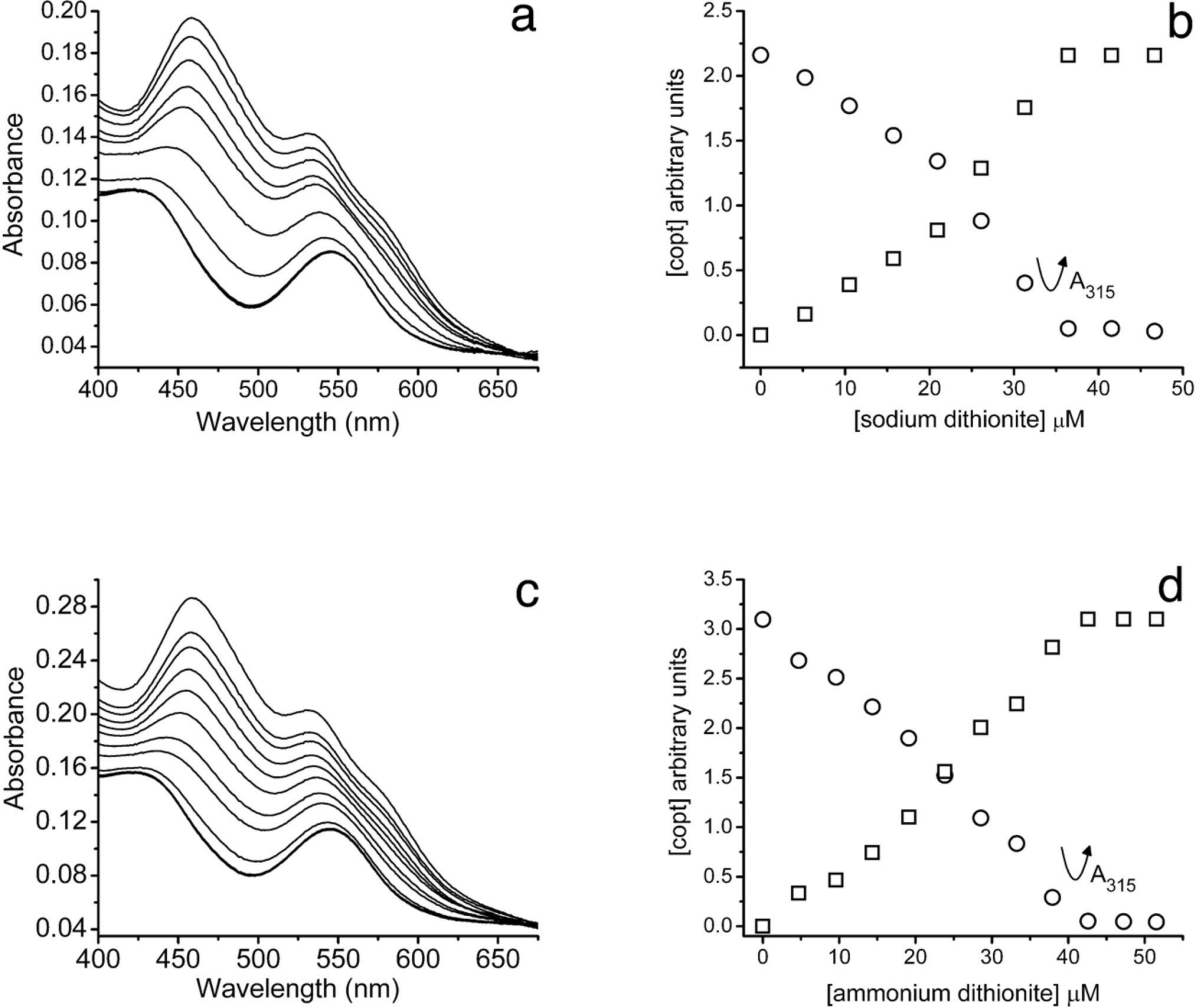
The anaerobic titration of the C-terminal domain of mitoNEET with sodium dithionite (**a**, **b**) and ammonium dithionite (**c**, **d**) in 10 mM ammonium acetate pH 8.5 using UV–visible spectroscopy. **a** Titration of mitoNEET (23.7 µM, 1.00 mL) with sodium dithionite (2.36 mM, serial additions of 2 µL, i.e., 0, 2, 4, 6, 8, 10, 12, 14, 16, 18 µL). **b** MCR-ALS optimization of the titration data (400–675 nm); [copt], optimized concentrations vs. concentration of dithionite added with the oxidized (open circles) and reduced (open squares) species normalized to the same intensities. **c** Titration of mitoNEET (34.5 µM, 1.00 mL) with ammonium dithionite (2.41 mM, serial additions of 2 µL, i.e., 0, 2, 4, 6, 8, 10, 12, 14, 16, 18, 20, 22 µL). **d** MCR-ALS optimization of the titration data 400–675 nm); [copt], optimized concentration vs. concentration of dithionite added with the oxidized (open circles) and reduced (open squares) species normalized to the same intensities. The additions of the dithionites when their excess became evident as an increase of absorbance at 315 nm are indicated

Incremental addition of either sodium or ammonium dithionite resulted in titration of the iron(III) in mitoNEET with an absorbance maximum at 457 nm as shown in Figs. 3a, c Because the UV–visible spectra for the oxidized and reduced forms of mitoNEET were strongly overlapping, the titrations were evaluated using multivariate curve resolution using alternating least squares (MCR-ALS) optimization [20]. In the figures, the scales were normalized such that the spectra for the oxidized and reduced species were of the same intensity. The optimized curves (Fig. 3b, d) were neither linear nor strictly stoichiometric. More than one reduction reaction could have taken place, albeit to a minor extent, or the reaction may have occurred in a stepwise fashion, e.g., a reduction in the dimer cluster followed by dissociation and reduction of a monomer cluster. These distinctions could not be made on the basis of currently available data. During the titrations, there was also a noticeable change in the absorbance at 315 nm, a decline in the value and a subsequent increase. This was consistent with the reduction of mitoNEET by dithionite followed by the appearance of an excess of the reagent. The point of inflection in each case also indicated a nearly stoichiometric reaction.

**Fig. 4:**
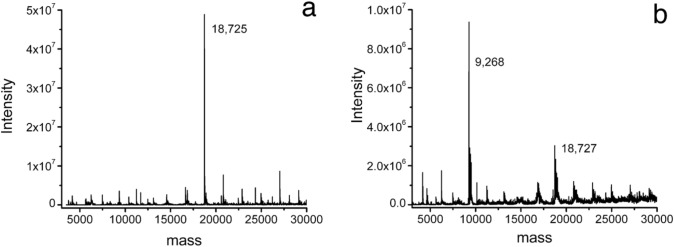
The anaerobic reduction of the C-terminal domain of mitoNEET with ammonium dithionite in 100 mM ammonium acetate pH 8.5 using ESI–MS. **a** Deconvolution of ESI mass spectrum of native C-terminal domain of mitoNEET (111 µM). **b** Deconvolution of ESI mass spectrum of native C-terminal domain of mitoNEET (111 µM, 3.00 mL) combined with ammonium dithionite (41.8 mM, 17 µL, ~ 2.1X)

When native samples of the version of mitoNEET currently under study (N-terminal Met-36) were subjected to native ESI–MS, the deconvoluted mass spectrum presented in Fig. 4a was typical. The samples of this native oxidized form of mitoNEET were analyzed on three separate occasions and gave an average deconvoluted value of 18,730 ± 5 Da. The calculated mass for the dimer with this sequence containing two 2Fe–2S (as [2Fe2S]^2+^) clusters [21] was 18,729 Da. A sample of the mitoNEET treated with ammonium dithionite (2.1X) was also subjected to native ESI–MS. The deconvoluted mass spectrum in Fig. 4b was obtained. The dissociation of the dimer upon reduction with dithionite was readily evident. The experimental mass of the monomer, 9268 Da, was inconsistent with retention of the FeS cluster. The most likely chemistry to account for the mass of the monomer would be the formation of S/O addition products. The calculated mass determined for the monomer (M) was 9190.590 Da whereas M + 2S + 1O was 9270.719 Da and M + 1S + 3O was 9270.653 Da. The difference of 0.066 Da was significantly less than our ability to resolve or reproduce masses experimentally, approximately 5 Da. Likewise, the experimental isotope distribution for individual ions, e.g. 8 + at m/z 1159, was only partially resolved and not satisfactory for isotopolog distribution calculations. An overinterpretation of the findings would posit that the lower calculated value (1S + 3O) would be closer to the experimental value, but only because the experimental values for the ions or the deconvolutions were all lower than corresponding calculated values. The adduct with –SO_3_ would still appear to be the most likely of the candidates. For example, the identified byproduct of the reaction of oxidized glutathione with sodium dithionite was glutathione-S-thiosulfate [19]. A comparison of the two dissociations was not clear cut. The chemistry was different, proton vs. electron transfer, as were the specific conditions, e.g. time and temperature. The dissociation of mitoNEET at pH 5.1 including loss of iron and sulfur was complete in about an hour on ice [13]. The anaerobic reduction followed by conveying the samples to the location of the mass spectrometer took much longer. None-the-less, it was noteworthy to find that dissociation of outer mitochondrial membrane protein mitoNEET dimers was promoted both by protons (pH) and electrons (redox), two integral components of the characteristic biological activity of the organelle. The findings also demonstrated that ammonium dithionite was compatible with redox chemistry investigations employing native ESI–MS measurements.

## Materials

Sulfur dioxide, greater than 99.9% Aldrich; acetic acid, 100% Honeywell Burdick and Jackson; ammonium hydroxide, ACS grade VWR Chemicals; methylene blue, Mallinckrodt Science Products; RIDOX Oxygen Scavenger, Fisher Scientific; Chelex 100 50–100 mesh, Sigma Life Sciences; L-glutathione reduced > 98%, Sigma Aldrich; L-glutathione oxidized 98%; Sigma Aldrich.

## Methods

### mitoNEET expression

The preparations of the cytosolic mitoNEET domain used previously [13] (beginning at Lys-32 with the starting Met for expression purposes corresponding to position-31) when expressed in C43-DE3 *E. coli* (OverExpress, BioSearch Technologies) developed a heterogeneity consistent with proteolysis at or near the N-terminus. Shifting the thrombin cleavable 6His tag to the N-terminus of this polypeptide did not alleviate the effect. Likewise using protease inhibitors and cocktails, different cells (NiCO21-DE3, New England Biolabs), or the TEV protease tag cleavage site did not overcome the heterogeneity [22]. The overall sequence was truncated to Lys-37 with the starting Met corresponding to position-36 in the mitoNEET sequence. With the 6His tag and thrombin cleavage site installed at the C-terminal as before via gene synthesis (Genscript), preparations of mitoNEET expressed in C43 cells were obtained with no evidence of proteolysis. The sodium ion adducts of mitoNEET in ESI–MS samples were persistent. The protein samples for mass spectrometry were dialyzed (6X, 10:1000) in plastic beakers against a solution of ammonium acetate (100 mM, pH 8.5). All aspects of the dialysis procedure were carried out in plastic containers and volumetric ware (graduated cylinders and pipette tips). The ammonium acetate solutions were prepared by dilution of 100% acetic acid to 100 mM and adjustment of the pH to 8.5 with concentrated ammonium hydroxide. The determination of the sodium content in the samples and their components were carried out by inductively coupled plasma mass spectrometry (ICP-MS). For example, after five repetitions of dialysis, the sodium concentration in the mitoNEET sample was 170 µM whereas the concentration in the dialysis buffer was less than 50 µM, almost all of it from the ammonium hydroxide used in its preparation. After six repetitions of dialysis, ions due to sodium adducts were still present, but at an adequately reduced level for the ESI–MS measurements.

### Experimental procedures

Redox reactions with salts of dithionite were carried out anaerobically in a glove bag containing argon that was passed through a gas washing bottle filled with activated RIDOX. The UV–visible spectrophotometer was inside the glove bag. The inert atmosphere was fortified by recirculation through a second gas washing bottle filled with activated RIDOX inside the bag using a battery powered aquarium air pump (Top Fin). Deoxygenation of mitoNEET solutions took place in a plastic beaker via argon purged dialysis inside the glove bag using Nordic ice packs lining a large crystallizing dish for cooling. Following dialysis, the mitoNEET solutions were transferred to a quartz cell for UV–visible spectrophotometry. Samples for analysis by mass spectrometry were kept in the UV–visible cuvette with a ground glass opening fitted with a ground glass stopper with an integral septum cap and sealed with Apiezon N grease. Inside the glove bag, the cuvette was placed in a round-bottom flask also sealed with grease in a ground glass joint for transfer to the mass spectrometer located on the Health Sciences Campus of the University of Toledo. The samples of the solutions for analysis were removed from the cuvette through the septum using a gas tight syringe (Hamilton). The temperature of the reduced reaction mixture was never higher than 10 °C and the pH of the solution following the mass spectrometric measurements was not lower than pH 8.1.

### ESI–MS

ESI–MS analyses were carried out with a Synapt G2-Si mass spectrometer (Waters) in positive ion mode. The solutions of the C-terminal domain of mitoNEET in ammonium acetate were infused into the ESI source at the flow rate of 10 µL/min using a syringe pump (Thermo). ESI capillary voltage was set to 2400 V, source temperature was 80 °C, and desolvation temperature was 120 °C. Sampling cone and source offset voltages were 20 and 80 V, respectively. Desolvation gas (nitrogen) flow rate was 300 L/h, and nebulizer gas setting was 6.1 Bar. Protein ions were analyzed by high resolution time of flight mode. ESI–MS spectra of oxidized mitoNEET were acquired in the 1000–3500 m/z range and in the 600–2500 m/z range for reduced protein using Mass Lynx (Waters) software prior to deconvolution with MaxEnt software.

ESI–MS analyses of solutions of oxidized and reduced glutathione in ammonium acetate as well as a solution of oxidized glutathione in the presence of ammonium dithionite were performed using a Synapt HDMS instrument (Waters) in positive ion mode. The solutions were infused into the nano-ESI source at the flow rate of 500 nL/min using a syringe pump. ESI capillary voltage was set to 3 kV, source temperature was 80 °C, and desolvation temperature was 150 °C. Sampling cone and extraction cone voltages were 45 and 1 V, respectively, trap collision energy (CE) was 6 eV, and transfer CE was 4 eV. Desolvation gas (nitrogen) flow rate was 500 L/h, and trap gas flow rate was 1.5 mL/min. ESI–MS spectra were acquired in 200–700 m/z range using Mass Lynx (Waters) software.

## Data Availability

The authors declare that the data supporting the findings of this study are available within the paper. Should any raw data files be needed in another format they are available from the corresponding author upon reasonable request.

## Abbreviations

FeS: Iron–sulfur
ESI–MS: Electrospray ionization mass spectrometry
VDAC-1: Voltage-dependent anion channel-1
EPR: Electron paramagnetic resonance
GSSG: Oxidized L-glutathione
GSH: Reduced L-glutathione
RP-HPLC: Reverse phase-high performance liquid chromatography
UV: Ultraviolet
MCR-ALS: Multivariate curve resolution- alternating least squares
ICP-MS: Inductively coupled plasma-mass spectrometry
